# Nutritional Status Has Marginal Influence on the Metabolism of Inorganic Arsenic in Pregnant Bangladeshi Women

**DOI:** 10.1289/ehp.10639

**Published:** 2007-11-19

**Authors:** Li Li, Eva-Charlotte Ekström, Walter Goessler, Bo Lönnerdal, Barbro Nermell, Mohammad Yunus, Anisur Rahman, Shams El Arifeen, Lars Åke Persson, Marie Vahter

**Affiliations:** 1 Institute of Environmental Medicine, Karolinska Institutet, Stockholm, Sweden; 2 International Maternal and Child Health, Uppsala University, Uppsala, Sweden; 3 Institute of Chemistry - Analytical Chemistry, Karl-Franzens University, Graz, Austria; 4 Department of Nutrition, University of California-Davis, Davis, California, USA; 5 ICDDR,B (International Centre for Diarrhoeal Disease Research, Bangladesh), Centre for Health and Population Research, Dhaka, Bangladesh

**Keywords:** arsenic, metabolite, methylation, nutrition, pregnant women, urine

## Abstract

**Background:**

The interindividual variation in metabolism of inorganic arsenic (iAs), involving methylation via one-carbon metabolism, has been well documented, but the reasons remain unclear.

**Objectives:**

In this population-based study we aimed to elucidate the effect of nutrition on As methylation among women in Matlab, Bangladesh, where people are chronically exposed to iAs via drinking water.

**Methods:**

We studied effects of macronutrient status using body mass index (BMI) among 442 women in early pregnancy (gestational week 8), and effects of micronutrient status (plasma folate, vitamin B_12_, zinc, ferritin, and selenium) among 753 women at gestational week 14. Arsenic metabolites in urine were measured by HPLC combined with hydride generation inductively coupled plasma mass spectrometry.

**Results:**

The median concentration of As in urine was 97 μg/L (range, 5–1,216 μg/L, adjusted by specific gravity). The average proportions of iAs, monomethylarsonic acid, and dimethylarsinic acid in urine in gestational week 8 were 15%, 11%, and 74%, respectively. Thus, the women had efficient As methylation in spite of being poorly nourished (one-third had BMIs < 18.5 kg/m^2^) and having elevated As exposure, both of which are known to decrease As methylation. The metabolism of iAs was only marginally influenced by micronutrient status, probably because women, especially in pregnancy and with low folate intake, have an efficient betaine-mediated remethylation of homocysteine, which is essential for an efficient As methylation.

**Conclusions:**

In spite of the high As exposure and prevalent malnutrition, overall As methylation in women in early pregnancy was remarkably efficient. The As exposure level had the greatest impact on As methylation among the studied factors.

Worldwide, millions of people are exposed to inorganic arsenic (iAs), a documented potent human toxicant and carcinogen, via drinking water [International Agency for Research on Cancer (IARC) 2004; World Health Organization (WHO)/International Programme on Chemical Safety 2001]. The British Geological Survey (BGS 2001) estimated that about 50 million people in Bangladesh alone are drinking water from tube wells that exceeds the WHO drinking-water guideline value for As of 10 μg/L. There is a marked variation in susceptibility to As, which—at least in part—may be mediated via variation in As metabolism (Vahter 2002).

iAs is metabolized by most mammals, including humans, via reduction and methylation reactions with *S*-adenosylmethionine (SAM) as the methyl donor (Hayakawa et al. 2005; Marafante and Vahter 1984; Vahter 2002). Dimethylarsinic acid (DMA) is the main As metabolite excreted in human urine, besides monomethylarsonic acid (MMA) and some remaining iAs, but there are major differences among individuals as well as between population groups (Vahter 2002). Usually, the proportions are 10–30% iAs, 10–20% MMA, and 60–80% DMA (Vahter 2002). The metabolism of As implies both detoxification and activation. The reduced trivalent forms, in particular MMA(III), are more toxic than the pentavalent forms (Bredfeldt et al. 2006; Schwerdtle et al. 2003; Styblo et al. 2002; Wang et al. 2007). A high concentration of MMA in the urine indicates a low capacity of further methylation to DMA and, probably, elevated concentrations of the highly toxic MMA(III) in the cells. There is increasing evidence of positive associations between urinary MMA and the prevalence of As-related bladder cancer (Chen et al. 2003b; Pu et al. 2007; Steinmaus et al. 2006), skin cancer (Chen et al. 2003a; Hsueh et al. 1997; Yu et al. 2000), other skin effects (Ahsan et al. 2007; Del Razo et al. 1997), structural chromosomal aberrations (Maki-Paakkanen et al. 1998), cardiovascular effects (Tseng et al. 2005), and increased retention of ingested As (Vahter 2002). Thus, it is essential to identify the mechanisms behind the wide interindividual variation in As metabolism.

Because As is methylated through one-carbon metabolism (Figure 1), it is likely that the availability of methyl groups via intake of protein (Lammon and Hood 2004; Marafante and Vahter 1984; Vahter and Marafante 1987) and other factors involved in the methylation cycles [e.g. folate and vitamin B_12_ (Spiegelstein et al. 2003; Spiegelstein et al. 2005)] are critical for As methylation. There is also experimental evidence for the involvement of essential trace elements such as selenium and zinc (De Kimpe et al. 1999; Hong et al. 2000; Walton et al. 2003), although the mechanisms are not clear. Because of the marked species differences in As methylation (Vahter 1999), it is difficult to extrapolate the results to humans. However, there is growing evidence for a nutritional regulation of As methylation in humans (Gamble et al. 2005, 2006, 2007; Heck et al. 2007; Steinmaus et al. 2005). In the present study, we aimed to elucidate the modifying effects of macronutrient status, assessed by body mass index [BMI; body weight (kg) ÷ height (m^2^)], and micronutrient status, assessed by biomarkers of folate, vitamin B_12_, Zn, ferritin, and Se status, on As metabolism in pregnant Bangladeshi women with a wide range of nutritional status and As exposure via drinking water.

## Methods

### Study area and population

Our ongoing research project concerning the effects of As exposure via drinking water on reproduction and child development is being carried out in Matlab, Bangladesh, where most of the approximately 200,000 inhabitants drink water from local tube wells, many of which contain high concentrations of naturally occurring As (Jakariya et al. 2005; Rahman et al. 2006b). Most of the tube wells were constructed in the 1970s and 1980s; thus, many of the younger residents have been exposed since birth.

This study on nutrition modification of As metabolism is nested into a population-based food and micronutrient supplementation trial [Maternal and Infant Nutrition Interventions of Matlab (MINIMat)] carried out by the International Center for Diarrhoeal Disease Research, Bangladesh (ICDDR,B) in Matlab, about 50 km southeast of Dhaka. This study includes approximately 4,500 women who enrolled in early pregnancy and were followed until 6 months postpartum. Pregnancy was identified by test of urine [gestational week (GW) 6–8] from women reporting amenorrhea at the time of the monthly visit by community health research workers within the Health and Demographic Surveillance System (HDSS) that ICDDR,B has been running in Matlab since the 1960s (Gwatkin et al. 2000). At the time of enrollment (around GW9), women were randomly assigned to be included in a community-based food supplementation program directly after confirmation of pregnancy by ultrasound (“early start”) or at the time of their own choice (“usual start”), about GW17. The supplemental food consisted of fried rice powder (80 g), fried pulse powder (40 g), molasses (20 g), and soybean oil (12 mL).

In the MINIMat trial, a total of 2,119 women were enrolled from January 2002 through December 2002. The loss of subjects from pregnancy testing until enrollment was about 29%; the main causes were refusal to participate, a false-positive pregnancy test, ineligibility for supplementation because of gestational age criteria (above GW13), early fetal loss or abortion, out-migration, or the woman could not be located. Women who were not enrolled were slightly older (by about 6 months) and had slightly lower socioeconomic status (SES) than the women who were enrolled. More details concerning background information, recruitment, and urine sampling have been described in a previous publication concerning the extent of As exposure (Vahter et al. 2006). We obtained SES from the HDSS.

One thousand of the women enrolled in the MINIM at trial between January and December 2002 (*n* = 2,119) were randomly selected for assessment of micronutrients in blood samples collected during GW14. This cohort of 1,000 women was also used for further analyses of As metabolites in urine. Although all women were included for analyses of the effects of micronutrients on As metabolism, a subset of the first 500 women selected was used for the assessment of the effect of macronutrient status. A selection scheme for the women studied is shown in Figure 2. For evaluation of the effect of macronutrient status assessed by BMI, which is often affected in early pregnancy, we used urine samples collected in GW8 on average. Of the first 500 randomly selected women, 442 had donated urine specimens and had their body weight and height measured at the time of pregnancy testing. Because of lack of data on protein intake in the studied women, we included urinary creatinine (U-Cre) concentrations as an additional proxy indicator of macronutrient status, as it is related to muscle mass and intake of meat (Suwazono et al. 2005). Although the gold standard is to measure creatinine in 24-hr urine, we adjusted the concentrations in our spot urine samples by specific gravity to compensate for variation in urine dilution.

For evaluation of the effect of micronutrient status, we used urine and blood samples collected in GW14, when the blood samples for measurements of nutritional markers were collected. Out of the 1,000 randomly selected pregnant women, 780 donated both blood and urine specimens. Twenty-seven urine samples in GW14 (but none in GW8) had a specific gravity of 1.001 g/mL, indicating highly diluted urine samples. Therefore, we excluded those samples (3.5% of the total number of 780 samples) from further analysis. Thus, we included 442 women in the macronutrient-effect analysis and 753 women in the micronutrient-effect analysis, with 341 women included in both.

### Determination of As metabolites

We assessed As metabolism using the percentages of iAs, MMA, and DMA in urine, as well as two methylation indexes [i.e., primary methylation index (PMI), defined as the ratio between MMA and iAs, and secondary methylation index (SMI), defined as the ratio between DMA and MMA. We used total urinary As [U-As; the sum of As metabolites (iAs + MMA + DMA) in urine] as the measure of iAs exposure. Concentrations of As metabolites in urine were measured with an Agilent 1100 HPLC system (Agilent Technologies, Waldbronn, Germany), coupled with hydride generation (HG) inductively coupled plasma mass spectrometry (ICPMS) (Agilent 7500ce series; Agilent Technologies, Japan). The method measures metabolites of iAs [iAs(III), iAs(V), MMA, and DMA], but not organic As species (e.g., arsenobetaine, arsenocholine) originating from the diet, because those arsenicals do not form volatile arsines (hydride generation) like iAs and its metabolites do. The method was described in detail by Lindberg et al. (2006). Determination limits were 0.1 μg/L for arsenite [As(III)] and MMA and 0.2 μg/L for DMA and arsenate [As(V)]. We adjusted As concentrations for variations in dilution by specific gravity (to the mean value of 1.012 g/mL); U-Cre, which is commonly used for dilution adjustment, was influenced more by age and nutrition than was specific gravity, and U-Cre was also associated with urinary As (Nermell et al. 2007).

For quality control purposes, we analyzed the reference material (CRM No. 18; National Institute for Environmental Studies, Ibaraki, Japan) with a certified DMA concentration of 36 ± 9 μg/L together with the collected urine samples. The concentration of DMA was 41 ± 3.4 μg/L (mean ± SD; *n* = 18). Urine samples were also analyzed for As metabolites by atomic fluorescence spectrometry (AFS) and for the sum of As metabolites by atomic absorption spectrometry (AAS), as reported by Lindberg et al. (2007a). The results of ICPMS and AFS showed good agreement for all metabolites; for iAs [As(III) + As(V)], MMA, and DMA, *R*^2^ = 0.91, 0.91, and 0.97, respectively (*n* = 221). Further, we obtained good agreement for the sum of As metabolites between the three different methods (for ICPMS vs. AFS, *R*^2^ = 0.97; for ICPMS vs. AAS, *R*^2^ = 0.96 (*n* = 221).

### Measurement of nutrient status

Venous blood samples were collected in GW14 at the Matlab health clinics, where they were kept at 6–8°C until transported (within 4 hr) to the hospital laboratory for separation of plasma, which was stored at −70°C until analysis of micronutrients at the University of California, Davis. We measured plasma ferritin (P-Ft) using radioimmunoassay (Diagnostic Products, San Diego, CA, USA), and plasma Zn (P-Zn) and plasma Se (P-Se) by AAS (Clegg et al. 1981). Ferritin values < 15 μg/L are usually considered to reflect low iron stores (WHO 2001). The cutoff level for Zn deficiency in pregnant women is 0.5 mg/L (Hotz et al. 2003), and the cutoff for low P-Se concentrations is 60 μg/L (0.75 μmol/L) (Van Dael and Deelstra 1993). Plasma folate (P-folate) and vitamin B_12_ (P-B12) were determined by the SimulTRAC-SNB radioassay kit (MP Biomedicals, Orangeburg, NY, USA). P-folate values < 4 μg/L (9 nmol/L) are considered indicative of folate deficiency (Gamble et al. 2006); the cutoff level for vitamin B_12_ deficiency is 0.25 μg/L (185 pmol/L) (Gamble et al. 2005). Urinary creatinine was analyzed by the Jaffé method.

### Statistical methods

We used Spearman’s rank correlation analysis to assess bivariate associations for identification of predictors of As metabolism and of potential confounding factors for further exploration in multivariate linear regression and analyses of covariance (ANCOVA). The tested potential confounding factors included As exposure (U-As), age, BMI, SES, parity, and GW. The factors with *p*-values < 0.1 in the bivariate tests were included in the further multivariate analyses. The exposure to As, age, SES, and BMI were considered the potential confounding factors; the final inclusion of confounders are noted in the tables. We used logarithmic transformation of variables when needed to meet the requirements of equal variances and to achieve approximately normal distribution. ANCOVA was used with categorical independent variables adjusted for continuous covariates for testing the difference in subgroups. A *p-*value < 0.05 was considered significant. For the statistical analyses, we used STATISTICA 7.1 (StatSoft Inc., Tulsa, OK, USA).

### Ethics

The study was approved by the Regional Ethical Committee at the Karolinska Institute and the Ethical Review Committee at ICDDR,B, Bangladesh. The study complies with applicable requirements of international ethical regulation. Informed consent was received prior to study start. The As-related ethical issues are described in more detail elsewhere (Vahter et al. 2006).

## Results

### Characteristics of the participants

The characteristics of the women are presented in Table 1. The 442 women included in the macro-nutrient study had their first urine samples collected in GW8 (range, 4.4–20 weeks; 10th/90th percentiles 5.9/11.4 weeks), whereas the 753 women in the micronutrient study had their second urine samples and blood collected in GW14 (range, 9.1–22 weeks; 10th/90th percentiles 12.5/16.5 weeks). The two groups were very similar with regard to recorded personal characteristics, except for body weight, which because of pregnancy was slightly higher in GW14. Mean age was 27 years (range, 14–44 years), with body weight of 45 kg (range, 30–72 kg) in GW8, height of 150 cm (range, 125–170 cm), and parity of 1.5 (range, 0–8 children); the women had on average 5 years of formal school education. Mean BMI was 19.9 kg/m^2^ (range, 13.9–32.0) in GW8 and 20.3 kg/m^2^ (range, 14.1–29.2 kg/m^2^) in GW14. The mean U-Cre concentration in GW8 was 0.57 g/L (range, 0.10–1.6 g/L), adjusted to an average specific gravity of 1.012 g/L. Mean (± SD) plasma concentrations were 5.1 ± 3.0 μg/L for P-folate, 0.23 ± 0.12 μg/L for P-B12, 0.61 ± 0.25 mg/L for P-Zn, and 35 ± 25 μg/L for P-Ft. About 60% of women were deficient in vitamin B_12_, 40% in folate, 30% in Zn, and 20% in iron (low ferritin). A subsample analyzed for P-Se showed a mean of 60 ± 11 μg/L (*n* = 89, randomly selected).

### Variation in As exposure and metabolism

The median concentration of total U-As in GW8 was 97 μg/L (mean, 159 μg/L), adjusted by specific gravity (1.012 g/mL), with a total range of 5–1,216 μg/L (10th percentile, 19 μg/L; 90th percentile, 385 μg/L). The mean ± SD proportions of iAs, MMA, and DMA in urine in GW8 were 15 ± 9.5%, (9.5), 11 ± 4.3%, and 74 ± 10.5%, respectively. We also found a considerable inter-individual variation in the distribution of the metabolites (Figure 3). The 10th and 90th percentiles were 7 and 24% for %iAs, 5 and 17% for %MMA, and 62 and 85% for %DMA. A similar variation in U-As metabolites was seen in GW14. The mean ± SD proportions of iAs, MMA, and DMA in urine in GW14 were 14 ± 6.9%, 8 ± 3.1%, and 79 ± 7.9%, respectively.

In the bivariate analysis intended for detection of predictors of As methylation and potential confounders, only As-exposure was markedly associated with percentages of iAs, MMA, DMA, and SMI (*p* < 0.001). Age was weakly associated with %iAs (*r_s_* = −0.09, *p* = 0.05 at GW8; *r_s_* = −0.08, *p* = 0.03 at GW14), SES with PMI (*r_s_* = 0.07, *p* = 0.1 at GW8; *r_s_* = 0.15, *p* < 0.001 at GW14), and BMI weakly associated with %DMA and SMI (*r_s_* = 0.06, *p* = 0.1 and *r_s_* = 0.05, *p* = 0.1, respectively at GW14). Because the As exposure level appeared to influence metabolism in a nonlinear way, as previously shown (Lindberg et al. 2008; Vahter 2002), we evaluated the distribution of the various metabolites by tertiles of U-As, representing low, medium, and high exposure (Table 2). The distribution of all the measured metabolites in urine in GW8 was highly influenced by U-As. After adjustment for age, BMI, and SES, the mean %iAs and %MMA increased and mean %DMA decreased significantly with increasing concentration of U-As (*p* < 0.01). Moreover, PMI was positively associated with the exposure (*p* < 0.05), whereas SMI was negatively associated with the exposure (*p* < 0.01). Essentially the same pattern was seen in GW14.

### Influence of macronutrient status on As metabolism

Because of the observed influence of the exposure to As on its metabolism, we adjusted for U-As in the evaluation of effects of macronutrient and micronutrient status on As methylation. A potential nonlinear relation between BMI (marker of macronutrient status) and As metabolites was evaluated by tertiles of U-As adjusting for age and SES. We found no significant association between BMI and the percentages of As metabolites or methylation indexes (*p* > 0.05). To test a potential linear relation between BMI and As metabolites (%iAs, %MMA, %DMA, PMI, or SMI) we applied multiple linear regression analyses, adjusting for U-As, age, and SES; this method also did not show any significant association. In contrast, U-Cre (adjusted by specific gravity) was significantly associated with most of the dependent variables when tested in a similar multiple linear regression analysis, despite a significant association between BMI and U-Cre (Spearman’s rank correlation tests, *r_s_* = 0.20, *p* < 0.001). With increasing U-Cre, the %iAs decreased (*p* < 0.01) and the %DMA (*R*^2^ = 0.19, *p* < 0.05), PMI, and SMI increased (*p* < 0.01).

### Influence of micronutrient status on As metabolism

Mean values of %iAs, %MMA, %DMA, PMI, and SMI were adjusted for age, BMI, and SES for different exposure levels (U-As stratified into tertiles); micronutrient indicators are shown in Table 3. The %iAs decreased with increasing P-folate concentration (13.4% in the first tertile of P-folate against 15.9% in the third tertile; *p* < 0.05) at the highest exposure level (third tertile of UAs; i.e., > 209 μg/L). No clear associations were seen between P-B12 or P-Ft and the percentages of As metabolites and methylation indexes, except for an indicated trend of decreasing %iAs with increasing level of P-B12 at the second tertile of exposure (58–209 μg/L). We found no overall association with P-Zn; however, at the highest As exposure level, %iAs and %MMA were higher and %DMA was lower at the highest Zn levels (*p* < 0.05).

Thirty-eight women had low folate, as well as low vitamin B_12_ and Zn (deficient group; all in the lowest tertiles: P-folate < 3.8 μg/L; P-B12 < 0.16 μg/L; P-Zn < 0.50 mg/L), whereas 36 women had adequate levels of all three micronutrients (adequate group; all in the highest tertiles: P-folate ≥ 5.3 μg/L; P-B12 ≥ 0.26 μg/L; P-Zn ≥ 0.64 mg/L). The deficient group had a slightly higher percentage of iAs in urine (14.3% vs. 12.0%; *p* < 0.05) and a lower PMI (0.43 vs. 0.61; *p* < 0.05) after controlling for age, BMI, and SES. The U-As (median values 96 and 126 μg/L, respectively), %MMA (6.2 and 7.4%, respectively), and %DMA (77.7 and 77.1%, respectively) were similar in deficient and adequate groups.

Multiple linear regression analysis, including U-As, age, BMI, and SES, showed that P-Se was not associated with any of the As metabolites or methylation indexes (*p* > 0.05; *n* = 89).

## Discussion

In contrast to the hypothesis, the results of this large population-based study show that the metabolism of iAs in rural Bangladeshi women in early pregnancy is only marginally influenced by nutritional status. In spite of the prevalent malnutrition, the Matlab women had a remarkably efficient methylation of As. The mean percentage of DMA (74%) in urine in GW8 was in the upper range of that observed in many populations in developed countries with much better nutritional status (Vahter 2002). Even in women deficient in folate, vitamin B_12_, and Zn, the As methylation efficiency was still very good, with > 75% DMA in the urine. The average proportions of the urinary metabolites of As (15, 11, and 74% for %iAs, %MMA, and %DMA, respectively) are similar to those reported for female subjects in Araihazar, a region approximately 30 km east of Dhaka, Bangladesh (Gamble et al. 2005); this shows that efficient As methylation is not a particular feature of the Matlab area.

**Figure 1:**
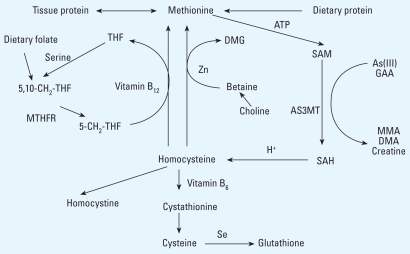
Overview of one-carbon metabolism and the methylation of As. Abbreviations: 5,10-CH_2_-THF, methylene tetrahydrofolate; AS3MT, As methyltransferase; DMG, dimethylglycine; GAA, guanidinoacetate; MTHFR, 5,10-CH_2_-THF reductase; SAH, *S*-adenosylhomocysteine; THF, tetrahydrofolate. Methionine, which originates from dietary protein and tissue protein, is activated by methionine adenosyltransferase to form SAM, which provides methyl groups for most methylation reactions in the body (e.g., methylation of DNA, GAA to creatine, and iAs to MMA and DMA). Methyltransferases are required for the methylation reactions, in which SAH is formed. SAH is hydrolyzed to homocysteine, which is used either for regeneration of methionine or for glutathione (GSH) biosynthesis in a transsulfuration pathway. In one methionine regeneration pathway, the methyl group is transferred to homocysteine from 5-CH_3_-THF and catalyzed by vitamin B_12_. In the alternate pathway, the methyl group is transferred from betaine and catalyzed by betaine:homocysteine methyltransferase (BHMT).

**Figure 2:**
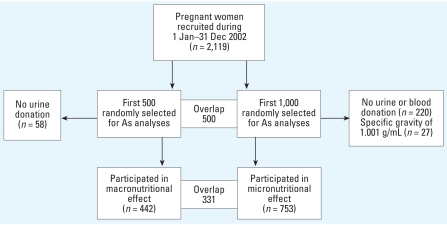
Selection of participants to studies of effects of macronutrient and micronutrient status on As metabolism.

**Figure 3:**
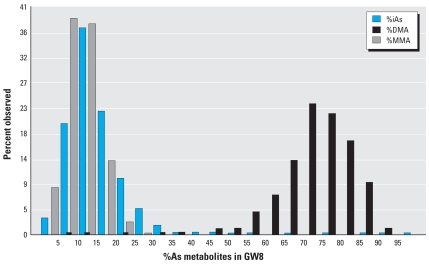
Frequency distribution of As metabolites in urine, demonstrating the interindividual variability in As metabolism among 442 pregnant women in GW8.

Previous studies in Bangladeshi men and women, however, showed a much stronger association (positive) between plasma folate and %DMA in urine (Gamble et al. 2005). In addition, folic acid supplementation caused a significant increase in urinary %DMA and a decrease in %MMA (Gamble et al. 2006). A likely reason for the lower nutritional influence on As metabolism in the present study is that women in childbearing age have a more efficient methylation of As than men (Lindberg et al. 2007b), particularly during pregnancy (Concha et al. 1998; Hopenhayn et al. 2003). This may be related to the *de novo* synthesis in women of choline by phosphatidylethanolamine methyltransferase (Vahter 2007), which is up-regulated by estrogen (Fischer et al. 2007). Choline is oxidized to betaine, which can donate its methyl group to homocysteine to form methionine (Figure 1). This reaction, catalyzed by betaine:homocysteine methyl-transferase, is the sole alternate route to the folate-dependent methionine synthase–catalyzed homocysteine remethylation (Ueland et al. 2005). Removal of homocysteine is a prerequisite for adequate one-carbon metabolism and As methylation (Figure 1) because the precursor to homocysteine, *S*-adenosylhomocysteine, is a strong feed-back inhibitor of the SAM-dependent methylation reactions, including the methylation of As (Marafante and Vahter 1984). Because there is a close interconnection between folate, vitamins B_12_ and B_6_, choline, betaine, and methionine in the human methylation pathway (Niculescu and Zeisel 2002; Stead et al. 2006; Ueland et al. 2005), the betaine-mediated remethylation of homocysteine is especially important in individuals with low folate intake, which was the case for many of the women in the present study. Gamble et al. did not report the effect of micronutrient status by sex, but they showed that women had higher %DMA than men (Gamble et al. 2005). We recently reported that BMI was positively associated with urinary %DMA and negatively associated with %MMA in men but not in women (Lindberg et al. 2007b); this supports our conclusion that women are less sensitive to poor nutrition for the methylation of As. This implies that women are also less susceptible to the health risks associated with high %MMA in urine, as discussed above. In fact, women seem to be less susceptible than men to As-induced skin effects (Rahman et al. 2006a; Vahter et al. 2007) and respiratory tract diseases (Smith et al. 2006; von Ehrenstein et al. 2005).

The fact that the women in the present study were pregnant also may have contributed to the marginal effect of nutrition on As methylation. In pregnancy, the endogenous synthesis of choline is particularly induced to meet the fetal demand of large amounts of choline for tissue growth and brain development (Zeisel 2006b), which may explain, at least in part, the increase in As methylation in pregnancy (Concha et al. 1998; Hopenhayn et al. 2003). The increase in plasma choline levels starts early in pregnancy and continues until term (Velzing-Aarts et al. 2005; Zeisel 2006a). Plasma betaine decreases from early in the first trimester until about GW20, and there is an increasing inverse betaine–homocysteine relation, emphasizing the importance of betaine in one-carbon metabolism in early pregnancy (Velzing-Aarts et al. 2005). Unfortunately, we did not measure plasma choline or betaine in the present study, but it seems likely that the betaine-mediated remethylation of homocysteine had started to increase, as many of the women were in their second trimester. There was an indication of higher %DMA in GW14 compared with GW8, and this will be further evaluated in a longitudinal study. An interesting parallel to the present results is the reported lack of changes in global liver DNA methylation in rat dams (and their fetuses) fed a folate-deficient diet with low methionine and choline content (Maloney et al. 2007). The folate deficiency was found to increase the liver choline at the expense of phosphocholine stores.

Of the factors evaluated in the present study, the As exposure level appeared to have the greatest impact on As methylation. With increasing exposure level, the proportion of iAs and MMA in urine increased and the proportion of DMA decreased, which is in line with previous *in vitro* studies (De Kimpe et al. 1999; Styblo et al. 2000) and epidemiologic studies (Hopenhayn-Rich et al. 1996; Lindberg et al. 2008). These results indicate that excess As inhibits the methyltransferases involved in As methylation, especially the methylation from MMA to DMA. We found that the increase in %MMA started at very low As exposure levels, corresponding to about 50 μg/L in urine; this is an important new finding because elevated MMA is a known risk factor for a wide range of As-related health effects (for review, see Tseng 2007).

Because of the lack of data on protein intake in the present study, we included U-Cre as a proxy indicator of macronutrient status because it is related to the muscle mass and meat intake (Suwazono et al. 2005). As expected, we found a significant correlation between U-Cre and BMI; U-Cre was much lower in the malnourished Matlab women (average, 0.57 g/L) than in populations with adequate nutritional status [median, 0.96 g/L in pregnant UK women during the entire gestation period (Waugh et al. 2003); 0.93 g/L in Swedish women during GW11–12 and 0.66 g/L during GW36–37 (Akesson et al. 2002)]. Interestingly, we found a positive association between U-Cre and %DMA, similar to findings in another recent study in Bangladesh (Gamble and Liu 2005). As we found no association between BMI and %DMA, we tend to believe that the correlation between U-Cre and %DMA is related to the fact that both U-Cre and DMA are products of the general methylation pathway using SAM as methyl donor. This was proposed by Gamble et al. (2005) in a study from Bangladesh in which urinary creatinine was the strongest predictor of As methylation, particularly for males. Methylation in the liver of guanidinoacetate to creatine, the precursor of creatinine, is the most important methylation reaction in the one-carbon methylation cycle from a quantitative point of view (Mudd and Poole 1975; Stead et al. 2006).

The main strength of this population-based study is the high number of women with a wide range of As exposures and nutritional status for which we were able to assess individual biomarker data, in combination with detailed data on anthropometry and SES. Further, the study population is fairly homogeneous, that is, rural Bangladeshi women in early pregnancy with essentially no smoking (< 1%) or alcohol consumption. In addition, the metabolism of As was assessed by the pattern of As metabolites in urine, determined by a sensitive HPLC-HG-ICPMS method. The limitations of the study include the wide range of GWs at the time of plasma and urine sampling, which may potentially influence the plasma concentrations of nutrients, especially folate and iron. However, we found no significant change in plasma folate by GW(< GW12.5, 4.3 μg/L; GW12.5–16.5, 4.5 μg/L; > GW16.5, 4.6 μg/L). Also, the likely start of some women in the food supplementation trial might have influenced their nutritional status. However, because we evaluated associations between biomarkers of As exposure and nutrient status and found very weak associations, this is not likely to have influenced the overall results. Another disadvantage is the limited amounts of collected plasma, which did not allow us to measure other micronutrients essential for one-carbon metabolism.

In conclusion, nutrition accounted for a minor part of the interindividual variation in As methylation in these highly exposed malnourished Bangladeshi women in early pregnancy. The average As methylation efficiency was unexpectedly high; we propose that this is related to the *de novo* synthesis of choline, which is essential for one-carbon metabolism and As methylation, especially in women with low folate intake and during pregnancy. Polymorphisms in As methyltransferases (e.g., *AS3MT*) and As(V) and MMA(V) reductases (e.g., *hGSTO1*) are likely to explain more of the observed variation in As metabolism, as found in our ongoing research involving population groups in Central Europe and Argentina (Lindberg et al. 2007b; Schlawicke Engstrom et al. 2007).

## Figures and Tables

**Table 1:** General characteristics of subjects in the macronutritional (GW8; *n* = 442) and micronutritional analyses (GW14; *n* = 753).

	Macronutritional analyses [No. (%)]	Micronutritional analyses [No. (%)]
Age (years)		
< 19.9	73 (16.5)	110 (14.6)
20–24.9	111 (25.1)	214 (28.4)
25–29.9	135 (30.5)	217 (28.8)
≥ 30	123 (27.8)	212 (28.2)
Unknown	0 (0.0)	0 (0.0)
Weight (kg)		
< 39.9	98 (22.2)	128 (17.0)
40–49.9	258 (58.4)	446 (59.2)
50–59.9	64 (14.5)	138 (18.3)
≥ 60	20 (4.5)	19 (2.5)
Unknown	2 (0.5)	22 (2.9)
BMI (kg/m^2^)		
< 18.5	135 (30.5)	179 (23.8)
18.5–25	281 (63.6)	521 (69.2)
> 25	24 (5.4)	31 (4.1)
Unknown	2 (0.5)	22 (2.9)
SES^a^		
Poorest	79 (17.9)	137 (18.2)
Second	94 (21.3)	174 (23.1)
Middle	103 (23.3)	153 (20.3)
Fourth	73 (16.5)	136 (18.1)
Richest	93 (21.0)	153 (20.3)
Unknown	0 (0.0)	0 (0.0)

a Quintiles of constructed wealth index based on asset ownership.

**Table 2:** Adjusted^a^ mean values of %iAs, %MMA, %DMA, PMI, and SMI stratified by urinary As (μg/L, tertiles) in GW8.

U-As (μg/L)	No.	%iAs	%MMA	%DMA	PMI	SMI
< 48	147	11.6	8.6	78.3	0.69	9.84
48–174	147	14.0	9.4	72.1	0.67	7.64
≥ 174	148	15.4	13.1	69.1	0.81	5.59
*p*-Value		< 0.01	< 0.01	< 0.01	< 0.05	< 0.01

a Using ANCOVA to analyze the effects of categorical independent variable of As exposure levels presented by tertiles of U-As and adjusted for age, SES, and BMI as continuous covariates.

**Table 3:** Adjusted^a^ mean values of %iAs, %MMA, %DMA, PMI, and SMI at GW14 stratified by U-As (tertiles), P-folate, P-B12, P-Zn, and P-Ft.

U-As (μg/L)	Micronutrients	No.	U-As (μg/L)	%iAs	%MMA	%DMA	PMI	SMI
P-folate (μg/L)								
< 58	< 3.8	76	32	10.8	5.9	81.7	0.54	13.91
	3.8–5.3	88	34	9.9	6.2	81.9	0.63	13.12
	> 5.3	81	32	10.0	6.8	81.0	0.69	11.87
58–209	< 3.8	86	117	12.9	7.0	77.3	0.54	11.07
	3.8–5.3	89	109	13.5	6.6	77.7	0.49	11.83
	> 5.3	80	108	11.8	6.5	78.8	0.55	12.07
> 209	< 3.8	78	360	15.9*	8.3	74.2	0.52	8.92
	3.8–5.3	85	370	14.1	8.1	75.4	0.58	9.28
	> 5.3	83	383	13.4*	7.8	75.9	0.58	9.72
P-B12 (μg/L)								
< 58	< 0.16	94	33	10.5	6.3	81.3	0.60	12.95
	0.16–0.26	80	34	10.7	6.1	81.1	0.56	13.41
	> 0.26	68	32	9.5	6.6	82.0	0.70	12.40
58–209	< 0.16	87	113	13.7	6.9	76.5	0.50	11.12
	0.16–0.26	82	110	12.7	6.7	78.2	0.53	11.60
	> 0.26	74	108	11.7	6.3	79.2	0.54	12.56
> 209	< 0.16	62	381	14.8	7.5	75.0	0.50	10.06
	0.16–0.26	81	355	14.8	8.3	74.7	0.56	8.97
	> 0.26	101	385	13.9	8.2	75.7	0.59	9.21
P-Zn (mg/L)								
< 58	< 0.50	79	35	10.5	6.2	81.1	0.58	13.17
	0.50–0.64	88	33	10.4	6.0	81.5	0.57	13.69
	> 0.64	84	31	9.7	6.9	81.9	0.71	11.90
58–209	< 0.50	97	112	13.4	6.7	76.9	0.50	11.47
	0.50–0.64	81	113	12.1	6.4	78.7	0.53	12.33
	> 0.64	72	110	12.6	7.0	78.3	0.56	11.18
> 209	< 0.50	73	373	14.4	7.4*	76.5*	0.51*	10.3*
	0.50–0.64	82	350	13.1*	8.2	76.7*	0.62*	9.37
	> 0.64	95	392	15.8*	8.5*	72.7*	0.54	8.5*
P-Ft (mg/L)								
< 58	< 21	81	31	10.1	6.3	81.7	0.62	13.03
	21–38	80	35	9.9	5.9	82.0	0.60	13.82
	> 38	85	33	10.4	6.8	81.2	0.65	12.01
58–209	< 21	92	115	12.5	7.2	78.4	0.57	10.90
	21–38	76	103	12.3	6.4	78.5	0.52	12.33
	> 38	82	116	13.4	6.5	76.7	0.48	11.83
> 209	< 21	76	339	14.3	7.5	76.6	0.52	10.23
	21–38	93	387	14.4	8.6	74.2	0.60	8.59
	> 38	82	390	14.8	8.1	74.9	0.55	9.29

a Adjusted for age, BMI, and SES.

* *p* < 0.05 between indicated tertiles of plasma nutrient levels.
